# Profiling of Indigenous Biosurfactant-Producing *Bacillus* Isolates in the Bioremediation of Soil Contaminated by Petroleum Products and Olive Oil

**DOI:** 10.1155/2021/9565930

**Published:** 2021-09-16

**Authors:** Paola Sandra Elenga-Wilson, Christian Aimé Kayath, Nicaise Saturnin Mokemiabeka, Stech Anomene Eckzechel Nzaou, Etienne Nguimbi, Gabriel Ahombo

**Affiliations:** Laboratoire de Biologie Cellulaire et Moléculaire (BCM), Faculté des Sciences et Techniques, Université Marien Ngouabi, BP 69, Brazzaville, Congo

## Abstract

Petroleum is, up to this date, an inimitable nonrenewable energy resource. Petroleum leakage, which arises during transport, storage, and refining, is the most important contaminant in the environment, as it produces harm to the surrounding ecosystem. Bioremediation is an efficient method used to treat petroleum hydrocarbon-contaminated soil using indigenous microorganisms. The degradation characteristics for a variety of hydrocarbons (hexane, benzene, gasoline, and diesel) were qualitatively and quantitatively investigated using *Bacillus* isolates. Microbiological and biochemical methods have been used including isolation of oil-degrading bacteria, enzymatic activities, the determination of physicochemical parameters, biosurfactant production and extraction assay, oil displacement assay, antimicrobial assay of the biosurfactants, and bioremediation kinetics. Consequently, of the 60 isolates capable of degrading different hydrocarbons at fast rates, 34 were suspected to be *Bacillus* isolates capable of growing in 24 h or 48 h on BH medium supplemented with 2% of hexane, benzene, gasoline, diesel, and olive oil, respectively. Among the 34 isolates, 61% (21/34) are capable of producing biosurfactant-like molecules by using gasoline, 70% (24/34) with diesel oil, 85% (29/34) with hexane, and 82% (28/34) with benzene. It was found that biosurfactant-producing isolates are extractable with HCl (100%), ammonium sulphate (95%), chloroform (95%), and ethanol (100%). Biosurfactants showed stability at 20°C, 37°C, 40°C, and 60°C. Biosurfactant secreted by *Bacillus* strains has shown an antagonistic effect in *Escherichia* coli, *Shigella flexneri* 5a M90T, and *Bacillus cereus*. The selected isolates could therefore be safely used for biodegradation. Substrate biodegradation patterns by individual isolates were found to significantly differ. The study shows that benzene was degraded faster, followed by hexane, gasoline, and finally diesel. The *Bacillus* consortium used can decrease hydrocarbon content from 195 to 112 (g/kg) in 15 days.

## 1. Introduction

The frequency at which industries have been exploiting the Earth's natural resources has left a colossal impact on the Earth's natural balance. Different commercial products like diesel, gasoline, hexane, and benzene-like molecules can cause soil pollution [1]. Soil contamination by organic hydrocarbons is one of the main reasons for environmental problems across the world because of their wide distribution, persistence, complex composition, and toxicity, leading to their accumulation in the environment to concentrations that may affect living beings. Physical, chemical, and biological techniques have been used for years to rehabilitate soils contaminated by those organic pollutants [2]. A number of physical and chemical methods are employed for the site remediation of contaminated soils [1]. However, these technologies are expensive and can lead to incomplete decomposition of contaminants [3]. One of the best approaches is bioremediation among different technologies for the clean-up of soil and groundwater [2]. It is an eco-friendly strategy and is mainly mediated through the natural soil microbial community. Biodegradation of organic waste has become an increasingly important method of waste treatment with main advantages including inexpensive equipment, environmentally friendly process, and simplicity. Certain microorganisms, mainly bacteria, have evolved to metabolize these contaminants by using them as nutrient and/or energy sources [4]. Bioremediation's efficiency depends on the presence of appropriate microorganisms and is also affected by the environmental conditions and microbial community composition. Bioremediation of polluted soils can be effectively done by using biosurfactant-producing microorganisms [5–8]. Biosurfactants are biological surface-active molecules with multiple applications in industries [3, 9–11]. They consist of two different parts as they are amphiphilic compounds that possess hydrophilic polar moiety and a nonpolar group which is hydrophobic. These properties enable them to reduce surface and interfacial tension and thus increase the surface area of the immiscible phases, increasing mobility, bioavailability, and subsequent biodegradation [12–14]. They are naturally occurring surfactants and have many advantages compared to their chemical versions. They are eco-friendly, biodegradable, and cheap and have minute toxicity. Bacteria belonging to genera *Bacillus* have been reported to produce various types of biosurfactants [4, 5, 10, 11, 15–18]. The genus *Bacillus* consists of a variety of species that display considerable biosurfactant production and produce cyclic lipopeptides and lipoproteins, including surfactins, fengycin, lichenysin, and bacillomycin as the major types of biosurfactants [19, 20]. Therefore, potential applications of microbial surfactants should be further exploited.

The Republic of Congo's economy is less diversified and mainly focused on hydrocarbon exploitation. Congo is one of the biggest petrol producers in sub-Saharan Africa with the production of 12 million tons per year and that production has not stopped increasing ever since. Hydrocarbons represent over 70% of primary energy consumption in Congo. Pollution of soils by hydrocarbons is a very huge problem as many industries do not use bioremediation to depollute those soils. Up to this date, no study has been done in Congo in order to assess the bioremediation potential of indigenous *Bacillus* species. The main objective of this study is to characterize *Bacillus* species profile in bioremediation and to assess biosurfactant-like molecules in the bioaugmentation.

## 2. Materials and Methods

### 2.1. Soil Sampling

The representative soil samples used in this study are soils contaminated for over 10 years by oil dirt. Samples were collected in four garages of different districts (Bacongo 4°17′14.3″S 15° 15′33.7″E, Ouenze 4°27′72.0″S 15° 15′25.17″E, Talangai 4°23′26.37″S 15° 29′44.95″E and Mfilou 4°24′45.47″S 15° 28′94.59″E) in Brazzaville. Soil samples of 1 kg were collected at three different points using a sterile shovel at a depth of 0–15 cm. These samples were put in polyethylene bags and were immediately conveyed to the laboratory for further analysis. Different soil parameters such as pH, electrical conductivity, total petroleum hydrocarbon (TPH) [21] content, and percent water moisture were evaluated.

The ability of the isolates to tolerate and/or to degrade hydrocarbon has been assessed as previously demonstrated [15]. Briefly, the Bushnell Hass (BH) medium has been used for enrichment and isolation of PAHs degrading bacteria. It contained (g/L) NaCl: 10.0 g, KCl: 0.29 g, MgSO_4_.7H2O: 0.42 g, KH_2_PO_4_: 0.83 g, NH_4_SO_4_: 0.42 g, K_2_HPO_4_: 1.25 g, and agar: 20 g. It was supplemented with 2% of gasoline, diesel fuel, benzene, hexane, or olive oil used individually as a sole source of carbon and energy. The medium was then autoclaved at 121°C for 15 min. Dilutions of 1 g of each wild contaminated soil sample were aseptically transferred into a sterile falcon tube containing 9 mL of sterile distilled water. Serial dilutions were done and the bacterial suspension was streaked on Bushnell Hass medium and incubated at 37°C for 2 days. Meanwhile, the inoculum was also streaked on the BH agar medium with no carbon source.

### 2.2. Isolation of Gram-Positive Oil-Degrading Bacteria

Bacterial species able to grow on BH agar medium supplemented with 2% of pollutants mentioned above were selected. Enumeration of selected colonies was done on Mossel agar medium supplemented with 4.2 mL of Polymixin B used to exclude Gram-negative bacteria. The plates were incubated at 37°C for 24 hours. Preselected isolates were repeatedly streaked on to Mossel agar plates in order to obtain a pure culture of bacteria and then maintained on slants of the same medium.

### 2.3. Characterization of Isolates

In order to characterize the isolates, most cultural and biochemical tests were performed by using microbiological and biochemical standard methods. The shape, size, and color of bacterial colonies were studied on Mossel agar plates after 24 hours of incubation. The morphological characterization has been done using a light microscope (OPTIKA, Italie). The Gram status of the bacterial isolates has been done using 3% of potassium hydroxide (KOH) [22]. A sporulation test was undergone to determine the ability of isolates to form endospores. Oxidase and catalase tests were as well conducted for all bacterial strains. Bacterial isolates were also tested for their ability to swarm, showing that most *Bacillus* species are motile.

### 2.4. Enzymatic Activities

*Bacillus* strains are known for their ability to degrade casein and starch. Casein hemolysis and amylolytic activities were evaluated for all isolates as previously demonstrated by Kayath et al. 2019 [23]. For the casein hemolysis activity, 1 g of agarose was put in an Erlenmeyer flask containing 100 mL PBS (Phosphate Buffer Saline). After heating the mixture until the agarose completely dissolved, it was cooled in a water bath at 50°C. 10 mL of skimmed milk was added. The mixture was poured into Petri dishes and let to solidify. Wells were aseptically made using sterile yellow tips. 50 *µ*L of supernatant of the overnight culture was deposited into the wells. After incubation at 37°C for 24 hours, the halo formed around the colony was measured. The tests were done in triplicate for each isolate.

On the other hand, for the amylolytic activity, a 24-hour aged colony was deposited on the surface of LB agar containing 1% starch. Petri dishes were incubated at 37°C for 24–72 hours. Iodine was used as a revelatory. The halo around the colony was measured [23].

In order to show that *Bacillus* isolates are capable of degrading lipids, three media were used. The first medium was LB agar supplemented with 1% olive oil. The second medium was LB agar with few drops of tween 20. The third medium used was Mossel supplemented with egg yolk. A fresh colony was deposited on the surface of each medium. The appearance of a halo around a colony was analyzed.

### 2.5. Detection of Biosurfactant Assay

All strains were tested for their ability to grow under different temperatures (20°C, 37°C, 40°C, and 60°C) using the BH medium supplemented with 2% of pollutants including individual gasoline, diesel, benzene, hexane, or olive oil. Isolates capable of growing on the different media were selected for the detection of biosurfactant assay.

#### 2.5.1. Microbial Growth on Hydrocarbons and Biodegradation

The growth kinetics of selected strains was done. Broth cultures were grown in duplicate in 100 mL flasks containing 50 mL BH medium with different hydrocarbons 2%: gasoline, diesel oil, benzene, hexane, and olive oil for 15 days [15]. Flasks were incubated on a rotary shaker at 37°C. Every second day, the optical density was measured at 600 nm (OD_600_) using a spectrophotometer (VIS SPECTRO PHOTO METER).

#### 2.5.2. Biosurfactant Production Assay

The emulsifying activity of a biosurfactant is its capability of retaining the emulsion of hydrocarbons or oils in water. This method is very important for industrial and environmental applications. 5 mL of an overnight culture were poured into a test tube containing 5 mL (v/v) of diesel fuel, gasoline, benzene hexane, and olive oil individually. The mixture was vigorously shaken for 3 min using a vortex mixer (VELP Scientifica, Italy). The tubes were then incubated at room temperature for 24 hours. The height of the emulsion layer and the total height of the mixture were then measured. The emulsification index (E24%) was calculated using the standard formula E24% =  (He /**Ht**) × 100, with He = Emulsion height, Ht = Total height of mixture, and E24% = Emulsification percentage after 24 h [24]. The stability of biosurfactants produced was evaluated at different temperatures (20°C, 40°C, and 60°C).

In addition, biosurfactant-producing capacity was found to be associated with the hemolytic activity. A colony was streaked onto a Columbia Blood agar medium. The Petri dishes were incubated at 37°C for 48–72 hours [25]. A positive test was evaluated by the presence of a clear zone around the colony. In this study *Bacillus subtilis* strain GL48 (Accession: MK099888.1) [15] has been used as a control.

#### 2.5.3. Oil Displacement Assay

The oil displacement assay was evaluated by measuring the diameter of the clear zones that occurred when a drop of a biosurfactant-containing solution is placed on an oil-water surface. The modified assay [26] was conducted as follows: 50 mL of distilled water were added to a large Petri dish (15 cm diameter). After this stage, 20 *µ*L of crude oil was added to the surface of the water, followed by the addition of 10 *µ*L of supernatant of the culture broth. The diameter of clear zones was determined. The test was done in triplicate for each selected strain.

#### 2.5.4. Biosurfactant Extraction

The biosurfactant extraction method developed by Kinouani Kinavouidi et al. 2020 has been performed in this study [27]. The extraction of the biosurfactant was conducted as follows: 1 mL of the inoculum was used to inoculate 100 mL of Luria broth medium in triplicate and incubated in a shaker incubator at 180 rpm at 37°C for 24 hours. Each culture was then centrifuged at 10,000 rpm for 20 min. After centrifugation, cell-free supernatants of each isolate were treated by acid precipitation adding drops of HCl continuously until reaching a pH 2. The supernatants were allowed to precipitate at 4°C overnight. Precipitation was collected by centrifugation at 10,000 rpm for 20 min. This method was repeated with alcohol and 40% ammonium sulphate. The granules obtained were weighed and used to evaluate their ability to emulsify (v/v) diesel oil, gasoline, hexane, and benzene separately. *Bacillus* isolates were further characterized by extraction using an organic solvent like chloroform.

#### 2.5.5. Antimicrobial Assay of the Biosurfactants

The antimicrobial activity of the produced biosurfactant extracted was tested against identified strains (*Escherichia coli*, *Shigella flexneri* 5a M90T, and *Bacillus cereus*) as recently demonstrated by Bokamba Moukala et al. 2020 [24]. The antimicrobial activity was evaluated by the agar disc diffusion method with some modifications. The antimicrobial activity was done on Luria Broth agar. Wells were aseptically prepared onto the gels. The microorganism to be tested was inoculated into the gel. A volume of 50 *µ*l of the biosurfactant solution was deposited onto wells. After an incubation period of 24 hours at 37°C, the diameter of inhibition zones was measured. The average of the three measurements was taken to ensure that the results were reproducible.

### 2.6. Biodegradation Kinetics of *Bacillus* Isolates Consortia

In order to determine the ability of the isolates to reduce the total hydrocarbons mentioned above, the total hydrocarbon was estimated at different periods by using wild contaminated soil. Before sterilizing the wild contaminated soil sample, the total hydrocarbon content has been evaluated. 1 kg of wild contaminated soil sample obtained from Bacongo garage was autoclaved for 15 min at 121°C. After cooling the soil at ambient temperature, the estimation of total hydrocarbon was evaluated. The best isolates were selected in terms of emulsification index and the growth rate in the presence of either benzene, hexane, diesel, gasoline, or olive oil. An overnight culture of the consortia of four *Bacillus* isolates M28, M29, ST70, and ST55_600nm_ was inoculated in the wild contaminated soil sample and incubated at ambient temperature for a period of 15 days. The wild contaminated soil without the selected bacteria was used as a negative control. Every five days, the total hydrocarbon in the soil was estimated.

### 2.7. Statistical Analysis

Statistical analysis results are presented as mean value ± standard deviation (SD). GraphPad Prism 7 was used for data analysis.

## 3. Results

### 3.1. Soil Parameters

The physicochemical parameters of the soil samples are evaluated and recorded in Table 1.

### 3.2. Characterization of Isolates and Microbial Growth Rates on Hydrocarbons

**Figure 1:**
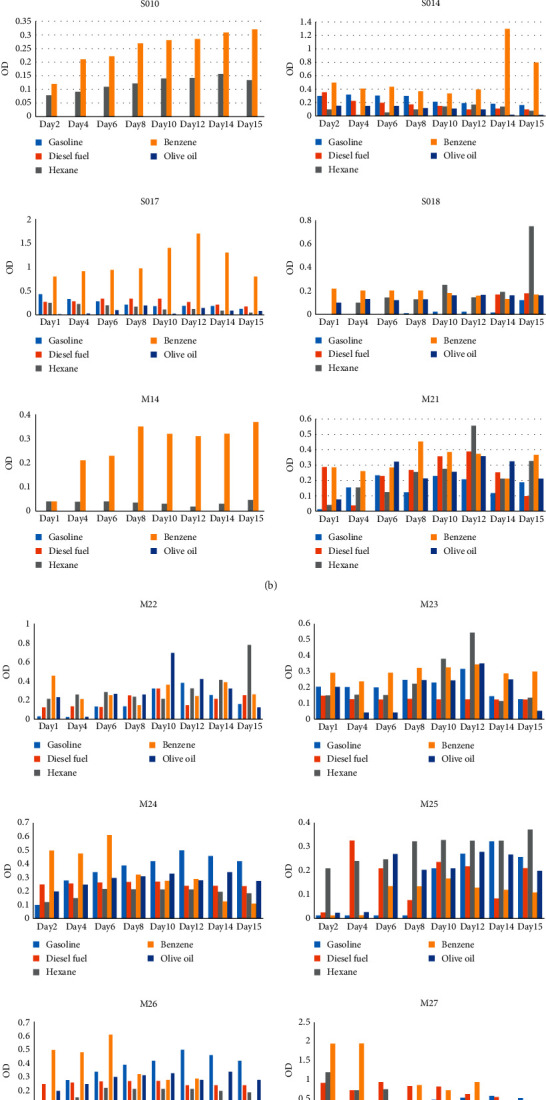
The growth of selected isolates on BH medium supplemented with 2% gasoline, diesel, benzene, hexane, and olive oil. OD _(600nm)_ is given in the *y*-axis, and the number of days is given in the *x*-axis. *Bacillus* species isolates are SB1, SB2, SB3, SB4, SB5, SB6, SB7, SB8, SO5, SO9, SO10, SO14, SO17, SO18, M14, M21, M22, M23, M24, M25, M26, M27, M28, M29, M30, M31, ST45, ST49, ST54, and ST70.

Effective biosurfactant-producing *Bacillus* species were isolated from contaminated soil samples in Brazzaville districts (Bacongo, Ouenze, Talangai, and Mfilou). 60 isolates were chosen based on their ability to grow on medium supplemented with 2% gasoline, diesel fuel, hexane, benzene, or olive oil in a period of fewer than 48 hours. According to Bergey's manual, the morphological and biochemical characterization of the isolates, 34 were studied and were suspected to be *Bacillus* species. The obtained isolates were examined to cultural characteristics on Mossel medium supplemented with polymixin B, microscopic examination (bacilli), endospore formation, a Gram-positive status with 3% KOH, and enzymatic activities. 69.7% of isolates were positive for the catalase test, 90.1% were able to hydrolyze casein, 84.8% were positive for starch hydrolysis, 78.78% for lipids hydrolysis, and 66.7% were positive for oxidase test. Furthermore, it was found that 93.9% of isolates were positive for the swarming test. All isolates tested were able to grow on the BH medium supplemented with 2% (gasoline, diesel, benzene, hexane, or olive oil) individually at different temperatures 20°C, 37°C, 40°C, and 60°C, respectively (Figure 1).

Specific growth rates of isolates on 2% gasoline, diesel, benzene, hexane, and olive oil individually were determined by linear relationship for optical density (OD) against time. Figure 1 illustrates the growth rates of the bacterial isolates cultivated in BH medium containing 2% concentration of gasoline, diesel, benzene, hexane, and olive oil individually as a sole source of carbon. The bacterial growth rate was observed for a period of 15 days (Figure 1).

### 3.3. Production of Biosurfactant-Like Molecules

**Figure 2:**
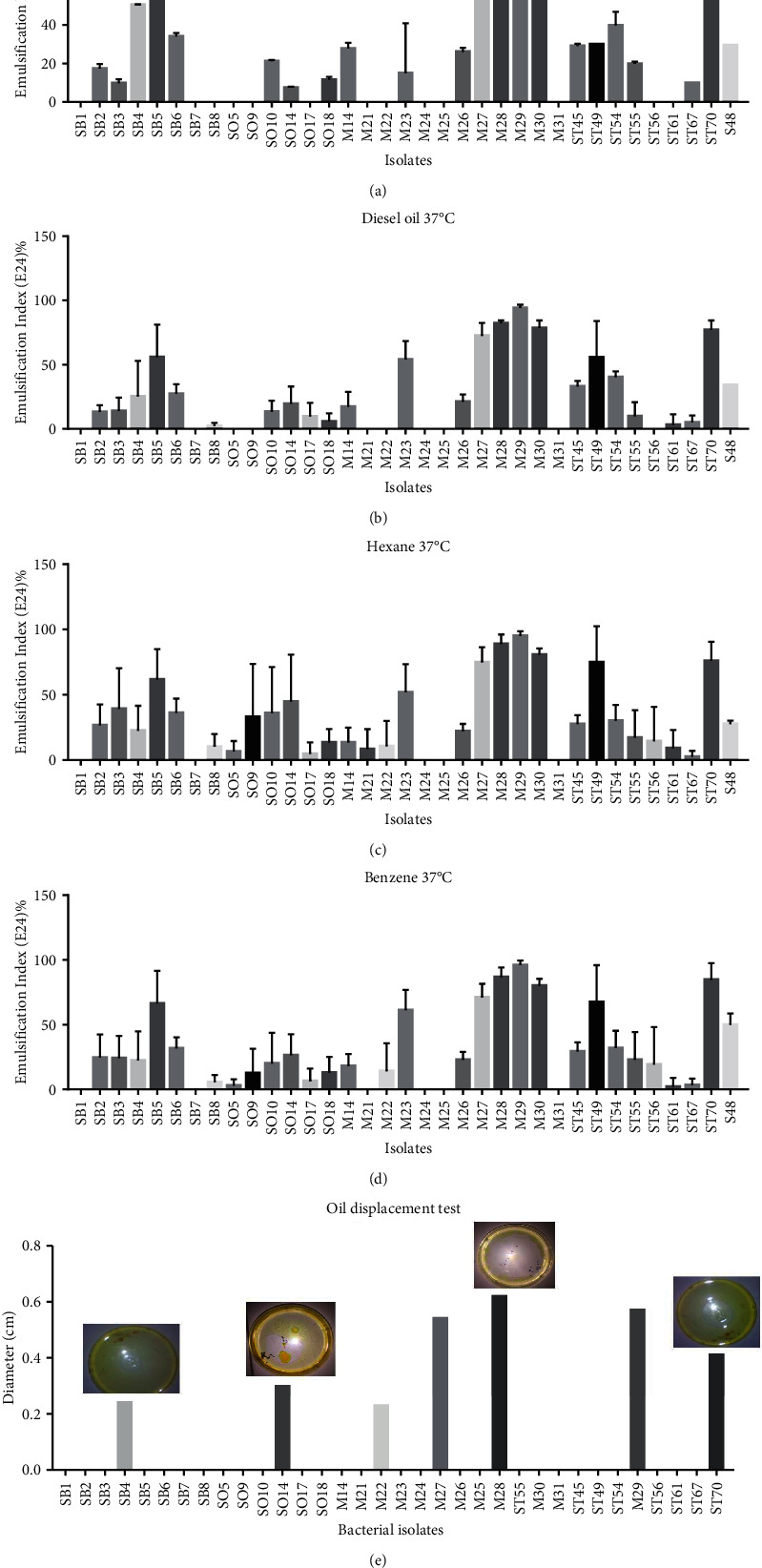
Emulsification activity of *Bacillus* isolates with gasoline (a), diesel oil (b), hexane (c), and benzene (d). Diameters of oil displacement test for the different isolates (e). Bacillus species isolates are SB1, SB2, SB3, SB4, SB5, SB6, SB7, SB8, SO5, SO9, SO10, SO14, SO17, SO18, M14, M21, M22, M23, M24, M25, M26, M27, M28, M29, M30, M31, ST45, ST49, ST54, ST55, ST56, ST61, ST67, and ST70.

All the three tests, including emulsification index (Figures 2(a)–2(d)) and oil displacement method (Figure 2(e)), used for the screening of biosurfactant producers showed the screened *Bacillus* species as effective biosurfactant producers. By using gasoline, diesel oil, hexane, and benzene; some strains were able to produce biosurfactant-like molecules with percentages ranging from 10 to 100% (Figures 2(a)–2(d)).

### 3.4. Effect of Temperature on Biosurfactants Activity

**Figure 3:**
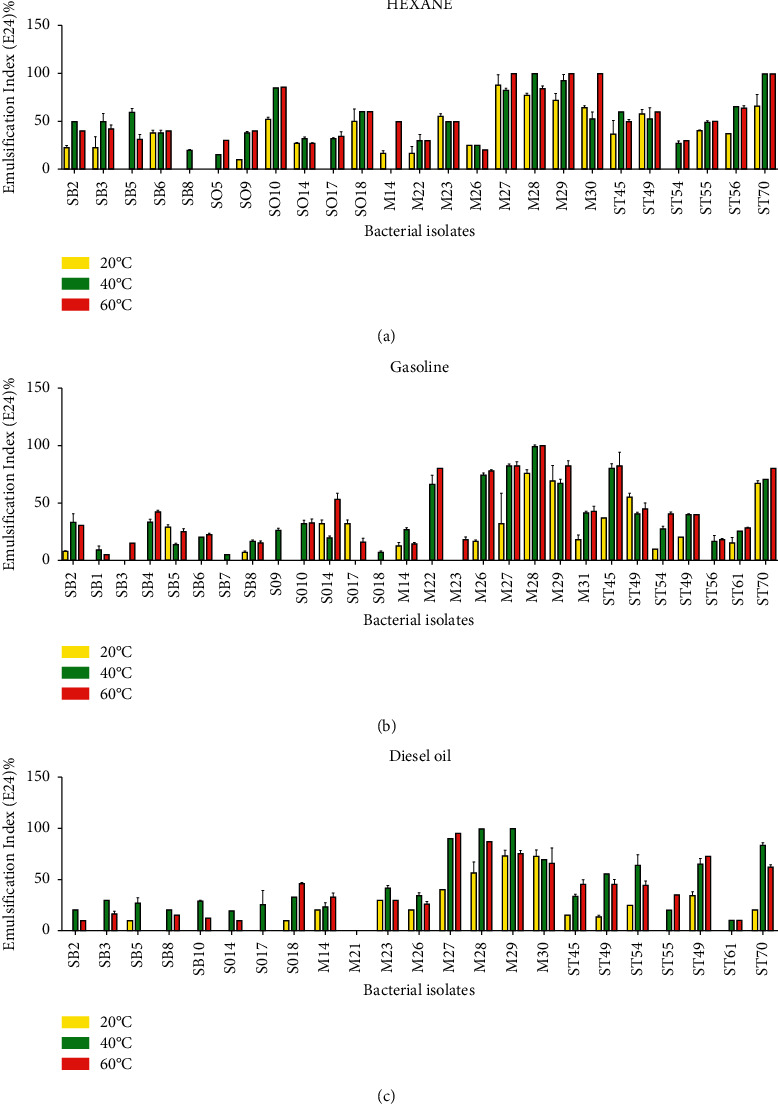
Emulsification activity of *Bacillus* isolates with hexane, gasoline, and diesel fuel at different temperatures. *Bacillus* species isolates are SB1, SB2, SB3, SB4, SB5, SB6, SB7, SB8, SO5, SO9, SO10, SO14, SO17, SO18, M14, M21, M22, M23, M24, M25, M26, M27, M28, M29, M30, M31, ST45, ST49, ST54, ST55, ST56, ST61, ST67, and ST70.

On average, isolates retained 60% of their activity at 20°C, 40°C, and 60°C for gasoline and diesel oil, respectively. On the other hand, some isolates lost their activity at 60°C. Incubation at room temperature for a period of 7 months had no significant impact on biosurfactants activity (Figures 3(a)–3(c)).

To highlight the extractable specifications of the biosurfactant-like molecule secreted by *Bacillus* species, strains with the ability to emulsify hydrocarbons (gasoline, diesel fuel, hexane, or benzene) have been performed for extraction. Precipitation on hydrochloric acid, ammonium sulphate, ethanol, and chloroform has been done. All assessed strains showed a precipitate except SB6 on chloroform and M14 on alcohol (Table 2). The emulsification index after precipitation has been carried on E24 (data not shown).

### 3.5. Antimicrobial Assay of the Biosurfactants

**Figure 4:**
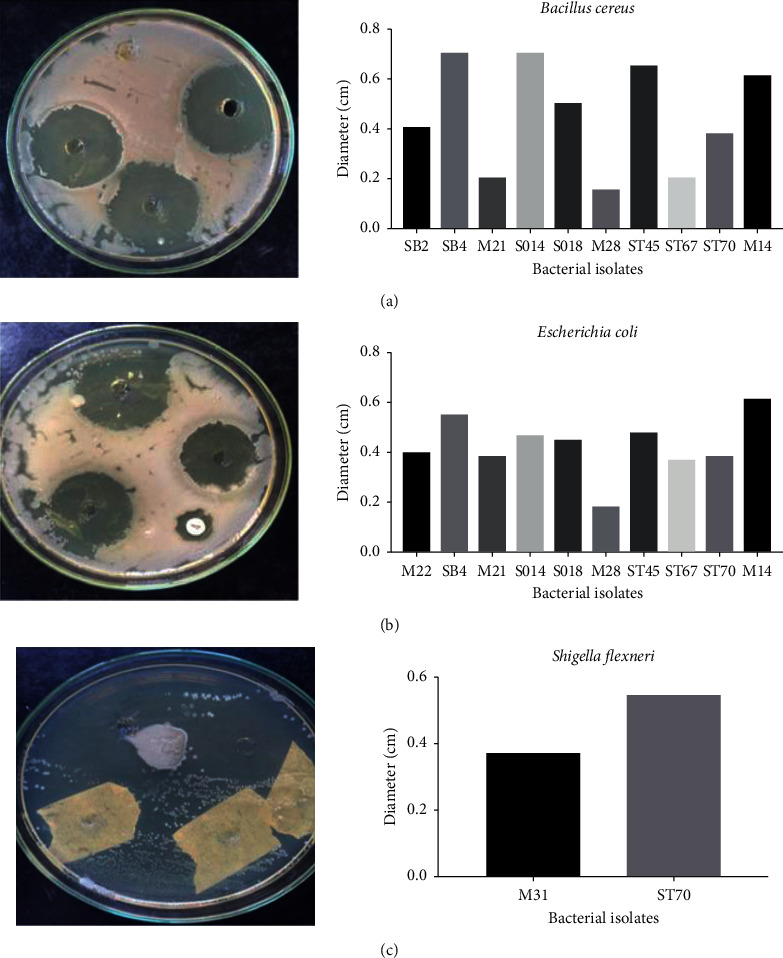
Antimicrobial assay of the biosurfactants against *Bacillus cereus* (a), *Escherichia coli* (b), and *Shigella flexneri* (c).

In order to assess the protective effect of biosurfactant with pathogens including *Shigella flexneri* 5a M90T, *Bacillus cereus,* and *Escherichia coli*, the antagonist assay has been done as explained in methods. Of the 34 isolates tested, 29.41% had antimicrobial activity against *Bacillus cereus* (Figure 4(a)), 34% against *Escherichia coli* (Figure 4(b)) and only 5.88% against *Shigella flexneri* 5a M90T (Figure 4(c)). The diameters of the halo varied from 0.7 to 6 mm from one isolate to another.

**Figure 5:**
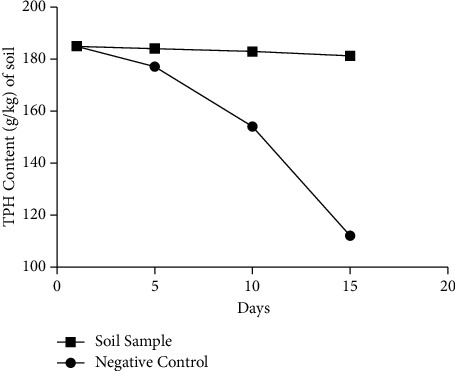
Biodegradation of *Bacillus* isolates M28, M29, ST70, and ST55 on contaminated soil.

A consortium of four isolates (M28, M29, ST70, and ST55) was used on contaminated soil in order to determine their ability to biodegrade hydrocarbons. It was found that the consortium could degrade hydrocarbons at 57.43% (Figure 5).

## 4. Discussion

The main objective of this study was to evaluate the biodegradation potential of autochthonous Bacillus species in soil depollution. A total of twelve soil samples obtained in four different districts of Brazzaville city (Bacongo, Ouenze, Talangai, and Mfilou) were characterized by different physicochemical properties. The results have shown that pH values of the different soils vary from 7.75 to 8.73 accordingly. The pH affects the solubility and bioavailability of soil constituents, which may affect biological activity in the soil. Soil samples obtained from garages in Ouenze are in some way neutral compared to those of Bacongo, Talangai, and Mfilou, which are more basic, respectively. Several studies have shown that a correlation between pH and microorganisms in bioremediation efficacy. The pentachlorophenol molecules can be biodegraded with the variation of pH and organic matter [28]. These were confirmed by the soil electrical conductivity measures [29]. Although it does not provide a direct measurement of specific ions or salt compounds, it is an important indicator as it influences microbial activity in the soil. Excess salts hinder growth by affecting water balance. Conductivity values obtained in this study ranged from 25 to 42 *µ*s/cm, corresponding to ISO11265:1994 standards of 30 to 60 *µ*s/cm for clay soils [29].

Many studies have been mentioned that the monitoring techniques of microbial hydrocarbon remediation in the soil seem to be issued to investigate [30–33]. *Bacillus* strains isolated in this work from different contaminated sites have been shown capable of degrading several pollutants such as polycyclic aromatic hydrocarbons. Microorganisms use petroleum hydrocarbons as a carbon for energy source. Bacteria belonging to the genus *Bacillus* are frequently involved in bioremediation and other biotechnological processes [10, 16, 17]. Many *Bacillus* strains including *B. subtilis*, *B. cereus*, have been shown capable of degrading complex hydrocarbon mixtures such as the biodegradation of crude oil [10, 16, 34–37]. *Bacillus* spp. are more tolerant to high concentrations of polycyclic aromatic hydrocarbons in soil due to their resistant endospores, so the isolates belonging to the *Bacillus* sp. could be effective in the removal of PAHs in the contaminated soils [2, 38–41]. The 34 *Bacillus* isolates in this study are able to grow in 24 hours or 48 hours on BH medium supplemented with different carbon sources (gasoline, diesel oil, benzene, hexane and olive oil) 2% (v/v). Most of these bacteria were not only able to grow in the presence of pollutants of hydrocarbons but also in BH medium supplemented with 2% olive oil. A study has shown that olive oil is an unfavorable substrate for microbial growth. Results show that these bacteria have an interesting biotechnological potential for industrial bioconversion of lipids and fats [42]. Microorganisms capable of degrading hydrocarbons exist in most environments. Their ability to degrade hydrocarbons has been demonstrated for large scale remediation. It has been reported that *Bacillus* sp. are among the most predominant isolates of crude oil [2, 40, 41]. The phenotypic and biochemical characteristics of the 34 isolates were related and oriented towards genus *Bacillus*. The sequencing of the 34 isolates associated *Bacillus* is the way for discrimination in terms of species and the amplification and sequencing of *fibE* are under investigation [23, 43].

The pattern of microbial growth differs from one organism to another. Factors that determine this microbial growth depend on incubation temperature, time, and the substrate used [1]. The growth pattern is reflected in the multiplication for the reported species. It has been reported that refined petroleum provides only carbon and energy to resident microorganisms while crude oil supplies nitrogen, sulphur, heavy metals, carbon, and energy [44]. The optical densities (OD600) show that bacterial species have a better growth in medium supplemented with benzene as carbon source followed by hexane, gasoline, diesel, and lastly, olive oil. Furthermore, the degradation rate of benzene and hexane is faster compared to that of diesel oil and gasoline, respectively. This may be explained by the complex structure and nature of commercial gasoline and diesel made of many aromatic rings.

Concurrent with the growth curve of each isolate at a specified time, their ability to emulsify hydrocarbons at different temperatures was investigated. It was found that a couple of bacterial isolates were able to grow on BH medium supplemented with different carbon sources but were negative for some biosurfactant screening methods. Except isolate ST70, all others did not show hemolytic activity. Similar results agree with our study as many investigators show hemolytic activity as an unreliable criterion for the detection of biosurfactant activity of a bacterial culture [4, 45].

Although seven isolates were found positive for oil displacement, numerous other isolates showed activity for emulsification with benzene, diesel, hexane and gasoline.

Many isolates in this study also showed a very fast growth rate and high growth capacity in the fact that they showed a very high exponential phase. The results provide evidence of the usefulness of *Bacillus* species for the bioremediation of contaminated environments. The growth rate in benzene and hexane is higher compared to that of gasoline and hexane. As evidenced, hexane and benzene are lighter and more volatile compared to gasoline and diesel. The ability of *Bacillus* species to survive in these oil products makes them very suitable for biodegradation of contaminated soils [10, 34, 36, 39].

This study shows that the production of biosurfactants depends on temperature and incubation period. It was shown that biosurfactants were able to precipitate alcohol (94.12%), ammonium sulphate (91.18%), hydrochloric acid (94.12%), and 94% were extractible by chloroform.

In order to show that these isolates can secrete biosurfactants able to inhibit pathogenic bacteria, biosurfactant-like molecules were tested against three known pathogenic isolates like *Shigella flexneri* 5a M90T, *Bacillus cereus,* and *Escherichia coli*. The study revealed that only biosurfactants produced from two (2) isolates were able to inhibit the growth of *Shigella flexneri*. Biosurfactants produced from ten (10) isolates inhibited the growth of *Bacillus cereus* and *Escherichia coli*. These results are very important to show not only their importance in biodegradation but also to avoid any contamination of the soil with these pathogenic bacteria. Bioremediation of polluted oily sludge employing pathogenic bacteria including *Pseudomonas aeruginosa*, *B. cereus*, *Staphylococcus* sp., has been demonstrated [36, 46, 47]. Here, we showed the evidence that *Bacillus* could be the bright choice for bioremediation since strains are deeply considered like GRAS (generally recognized as safe) organisms.

The total petroleum hydrocarbon content of contaminated soil obtained from Bacongo has decreased from 195 to 112 (g/kg) in 15 days. Though the biodegradation process is occurring at a slow pace, this shows that the bacterial consortium is able to degrade different hydrocarbons present. The bioremediation using consortia has been demonstrated [38, 48–51]. This process would require more conditions to be optimized for faster degradation.

## 5. Conclusions

This study has revealed that autochthonous *Bacillus* species isolated in the Republic of Congo are effective biosurfactant producers as they can grow very fast in media with either benzene, hexane, gasoline, diesel, or olive oil as the sole source of carbon individually. The consortium of four *Bacillus* isolates showed that these microorganisms presented great potential for the biotechnological and bioremediation of contaminated soils.

## Figures and Tables

**Table 1:** Results of pH, conductivity, water percent and TPH content.

Districts	Soil samples	pH	Conductivity (*µ*s/cm)	Water percent (g/L)	TPH content (g/kg) of soil
Bacongo	*B*_1_	8.50 ± 0.25	38.46 ± 0.35	47.31 ± 1.8	195.9
	*B*_2_	8.20 ± 0.16	33.86 ± 1.22	42.66 ± 2.05	190
	*B*_3_	7.90 ± 0.24	37.42 ± 1.36	46.00 ± 0.01	187

Ouenze	*O*_1_	7.20 ± 0.25	27.0 ± 0.4	40.00 ± 0.21	112
	*O*_2_	6.80 ± 0.45	23.00 ± 0.28	35.00 ± 0.02	142
	*O*_3_	7.75 ± 0.37	25.73 ± 0.51	41.00 ± 0.81	128

Talangai	*T*_1_	8.73 ± 0.33	42.37 ± 0.24	64.00 ± 2.94	141
	*T*_2_	8.67 ± 0.32	43.42 ± 0.12	63.00 ± 0.28	152
	*T*_3_	8.60 ± 0.31	40.00 ± 0.25	60.00 ± 0.30	127

Mfilou	*M*_1_	8.30 ± 0.32	31.90 ± 0.93	41.00 ± 0.47	139
	*M*_2_	8.3 ± 0.0	29.40 ± 0.32	42.00 ± 0.34	154
	*M*_3_	8.60 ± 0.54	25.0 ± 1.2	39.00 ± 0.37	126

**Table 2:** Biosurfactants profiles secreted by *Bacillus* sp.

Isolates	HCl precipitation	Ammonium sulphate precipitation	Alcohol precipitation	Chloroform extraction
SB2	+	+	+	+
SB3	+	+	+	+
SB4	+	+	+	+
SB5	+	+	+	+
SB6	+	+	+	+
SO10	+	+	+	+
SO14	+	+	+	+
SO18	+	+	+	+
M14	+	+	−	+
M23	+	+	+	+
M26	+	+	+	+
M27	+	+	+	+
M28	+	+	+	+
M29	+	+	+	+
M30	+	+	+	+
ST45	+	+	+	+
ST49	+	+	+	+
ST54	+	+	+	+
ST55	+	+	+	+
ST67	+	+	+	+
ST70	+	+	+	+

## Data Availability

The Excel sheets including the data used to support the findings of this study are available from the corresponding author upon request.
